# Cross-national variation in faith sharing across religious traditions

**DOI:** 10.1038/s41598-024-83531-z

**Published:** 2025-04-30

**Authors:** Robert D. Woodberry, Matt Bradshaw, Tyler J. Vander Weele, Byron R. Johnson

**Affiliations:** 1 Institute for Studies of Religion, Baylor University, One Bear Place 97236, Waco, TX 76798 USA; 2 Department of Biostatistics, Harvard T.H. Chan School of Public Health, Boston, MA USA; 3 Department of Epidemiology, Harvard T.H. Chan School of Public Health, Boston, MA USA; 4 Human Flourishing Program, Institute for Quantitative Social Science, Harvard University, Cambridge, MA USA; 5 School of Public Policy, Pepperdine University, Malibu, CA USA

**Keywords:** Religious dialogue, Evangelism, Conversion, Proselytism, Cross-national, Religious change, Evolution, Risk factors

## Abstract

Who shares their faith with people who have different religious views? This article examines ‘faith sharing’ across 22 religiously diverse countries. To our knowledge, this is the first study of ‘faith sharing’ across national and religious contexts. Evangelism can be controversial, but both historically and in our data, it is widespread—including in societies that restrict religious liberty. ‘Faith sharing’ is lowest in Europe, Japan, Australia, and the US and highest in Africa, Latin America, the Middle East, and Asia (excluding Japan). Within countries, there is no consistent pattern about which religious groups share their faith more. Previous research about ‘faith sharing’ focuses on Christians in the US, but the factors that predict ‘faith sharing’ in the US often do not generalize. In fact, the association with age, education, marital status, employment status, and immigration status is exactly the opposite in many societies. Because past comparative research ignores this topic and because so much of what we think we know about faith sharing is based on US Christians, we hope this article will spur further research and expand our understanding of religious persuasion and global religious change.

## Introduction

Religious change is widespread. In the past two centuries, and especially since World War II, the world’s religious landscape has shifted radically. Christianity has spread in Asia, Secularism in Europe, both Islam and Christianity in Africa, Protestantism in Latin America, and Hinduism, Buddhism, and Islam in Europe, North America and the Caribbean—some changes are via migration, but others via conversion^1–4^. New versions of Buddhism, Hinduism, Islam and Christianity developed and spread globally. Hinduism, Buddhism, Islam, and Christianity have often spread at the expense of ‘oral’ religious traditions, although these traditions often persist as folk traditions within global religions^4–8^. Yoga and meditation, which emerged as *lay practices* among Neo-Buddhists and Neo-Hindus in the late nineteenth and twentieth century via competition with Christians, have become widespread even among those who do not claim a Buddhist, Hindu, or even religious identity^9–13^.

We know these changes are happening and we can document changes that happen via migration and differential fertility, but we know little about who spreads these beliefs, behaviours, and identities. In response to conversions, some institute anti-conversion laws or spur violence. Yet, while strong responses to proselytism and conversion grab attention, much proselytism is likely prosaic: people sharing something they value with friends, and friends either coming to agree or retaining their old views. These interactions may or may not strain relationships. Regrettably, we have little empirical data about these interactions. Longitudinal surveys reveal religious changes, but we typically do not know what role others played in the process.

Most discussions of conversion, evangelism, and missions focus on Christianity. Scholars often recognize Islam and Buddhism as missionary religions, but many think few proselytize currently. However, all major religions have historically converted others and most continue to now, e.g., Muslims ^14,15^, Buddhists ^1,16–18^, Hindus ^19–23^, Jews ^24–27^, and others^4^.

In fact, no beliefs, practices, or identities (whether secular or religious) would be widespread without ‘converting’ others at some point. Once established, traditions can spread through childbirth and retention, if adherents have more children than others. However, non-religious people typically have fewer children than religious people, thus spreading non-religious views depends disproportionately on ‘persuasion’.

Because we lack good data, we know almost nothing about who shares their faith or how it influences others (or themselves). Moreover, what we know about ‘faith sharing’ is almost entirely about Christians in the US. After extensive searching we found several nationally representative surveys that ask related questions: i.e., Is proselytism ‘OK?’, or is it ‘important?’, ‘Have you been on short-term mission trips?’, and ‘How often do you share your faith with others?’. We also identified one cross-national survey that asked Muslims if they have a duty to convert others. Thus, the cross-national, multi-religious Global Flourishing Study (GFS) data presented here is both unique and valuable.

### Is it OK to evangelize others?

Some surveys ask if it is ‘morally OK’ to try to convert others. In the US, 49% of 18–23 years-olds on Wave 3 the *National Survey of Youth and Religion* (NSYR) say it is OK to try to convert others, but Conservative Protestants (CPs), Black Protestants (BPs), and Mormons (LDS) had more positive attitudes than others. Only 19% of Jewish young adults say it is OK^28^. Moreover, if we compare Wave 1 with Wave 3, respondents became slightly more averse to proselytism over time. This trend is particularly strong among Mainline Protestants and Roman Catholics^28,29^.

Internationally, there is very little data about people’s attitudes about evangelism, missions, and conversion—although it is likely that people have more positive views of converting people *to* their religion, than converting people *from* their religion.

### Is it important to evangelize others?

In 2007, the Baylor Religion Survey asked US respondents “if one wishes to be a good person” is it important to “convert others to your religious faith?”, 13.4% said ‘very important, and 19.9% said ‘somewhat important,’ for a total of 33.3%.

### Short-term missions

Other surveys ask respondents if they have gone on short-term missions. Millions of North Americans have, perhaps as many as 1.5 million go each year^30,31^. Fortunately, one national representative survey asks respondents about mission trips: the [US] National Study of Youth and Religion (NSYR). According to the NSYR, 30% of 13–17 years olds in the US have been on a ‘missions or religious service’ trip. This is particularly true for Mormons (70%) and Mainline Protestants (45%)^29^. Five years later, the same students were asked if they had participated in a ‘missions or religious service trip’ in the past two years: 15% said that they had: especially Mormons (35%) and Conservative Protestants (CPs: 22%)^28^.

### How often do you share your faith?

Other surveys ask respondents how often they share their faith. However, most *only ask religious people* if they share their faith; they do not ask *non-religious people* if they share their non-religious beliefs with others. Surveys also seldom ask if people try to change other’s ‘non-religious’ beliefs and identities, e.g., trying to change others’ political ideologies. Yet, it is unclear why trying to convince someone to become a Marxist, Democrat, Republican, or gender-role equalitarian is distinct from trying to convince someone to become Presbyterian. Moreover, without good survey evidence we do not know what types of beliefs and identities people try to ‘convert’ others to, nor even which groups are most active in trying to change other’s beliefs and identities.

We found one survey that asked ‘non-religious people’ about their proselytism. The 2007 *U.S. Religious Landscape Survey* asked ‘atheists, agnostics, and the non-religious’ “How often do you share your views on God and religion with religious people?” 12.8% said ‘at least one a week’ and 10.5% ‘once or twice a month.’ Only 32.4% said they ‘never’ do. Thus, the ‘non-religious’ report sharing their beliefs about ‘God and religion’ with religious people almost as often as religious people report sharing their views of God with non-believers or people from other religious backgrounds.

This reveals that in the US, sharing about one’s religious beliefs is relatively common for both religious and non-religious people. Some religious groups report sharing their faith more than others (e.g., CPs, BPs, and LDS share more than Mainline Protestants, Catholics and Jews). Protestants and LDS are also more involved in organized ‘faith-sharing’ activities. However, we do not know how much attempting to change others’ religion, compares to attempting to change other beliefs, behaviors, and identities.

### Expanding beyond the US

Among Christians, about half of full-time, cross-cultural missionaries are *from* Africa, Asia, Latin America, and Oceania and about half from Europe, North America, Australia, and New Zealand^8^. Surveys that ask about ‘faith sharing’ outside the US are extremely rare. However, the Pew Research Center’s survey “The World’s Muslims” (2008–2012) does. It is a stratified probability sample of 39 countries, including every country with more than 10 million Muslims, except for China, India, Saudi Arabia, and Syria, which Pew skipped because of political and security concerns^32^. In these 39 countries, 60.1% of Muslims believe “[they] have a duty to try and convert others to [their] religious faith [Islam]”. This percent is higher than the 58.2% of Muslims who favor making sharia “the official law of the land”.

Direct comparisons are difficult, because the questions on US surveys are different from the Pew Forum’s survey of Muslims. But a higher percentage of Muslims believe it is their “duty to convert others to Islam” than the percentage of people in the US that feel trying to convert others is ‘important’ (60% vs. 33%) or that think trying to convert others is ‘morally OK’ (49%). Moreover, conversion rates from other religions to Islam are high^33^ and there are now Muslim communities among Aborigines in Australia, Mayans in Mexico, and in other communities which historically had no contact with Islam^4^.

Other nationally representative survey data about proselytism by non-Christian religious traditions outside the US are lacking. Thus, while it is clear that most traditions have sought and still seek new followers, it is unclear what the relative prevalence of proselytism and other forms of faith sharing are. To our knowledge, the Global Flourishing Survey (GFS) allows us to compare ‘faith sharing’ between different religious traditions and countries around the globe for the first time.

### Limitations of previous research

As mentioned previously, there is almost no academic research about ‘faith sharing’ and what exists focuses almost exclusively on Christians in the US. Although the Pew survey of Muslims allows rough comparisons between Global Muslims and U.S. Christians, different question wording prevents direct comparison. We know almost nothing about Buddhists, Hindus, Jews, or non-US Christians who share their faiths.

Moreover, most research focuses entirely on whether *religious people* share their faith. Most surveys do not even ask *non-religious people* if they share their beliefs with others. However, it is not clear why convincing someone to stop believing in God is different from convincing someone either to start believing in God, or to change their view of God.

This study has certain limitations, documented below, but asks representative samples from a diverse set of countries and from all major religion/non-religious traditions whether they “tell others about [their] religious or spiritual beliefs even when they have different beliefs”. There is some ambiguity about what this ‘faith sharing’ entails, but multiple comparisons between the GFS questions and questions that ask directly about conversion give similar percentages. These comparable percentages suggest that a high proportion of people affirming the GFS question are trying to persuade others (see the Measures section). This article also demonstrates that faith sharing is widespread in every major religious group.

## Methods

The description of the methods below was adapted from Vander Weele et al. (2024)^34^. Further methodological details are available elsewhere^35–40^.

### Study population

The Global Flourishing Study (GFS) is a study of 202,898 participants from 22 geographically and culturally diverse countries, with nationally representative sampling within each country. It focuses on the distribution of determinants of well-being. Wave 1 of the data included the following countries and territories: Argentina, Australia, Brazil, Egypt, Germany, Hong Kong, India, Indonesia, Israel, Japan, Kenya, Mexico, Nigeria, the Philippines, Poland, South Africa, Spain, Sweden, Tanzania, Turkey, United Kingdom, and the United States. The countries were selected to (a) maximize coverage of the world’s population, (b) ensure geographic, cultural, and religious diversity, and (c) prioritize feasibility and leverage existing data collection infrastructure. Gallup Inc. collected the data. Most Wave 1 data were collected in 2023, although in some countries data collection began in 2022. Exact dates varying by country^38^. Four additional waves of panel data on the participants will be collected annually from 2024 to 2027.

The sample was designed to ensure nationally representative samples for each of the 21 countries and 1 territory (Hong Kong). Further details are available in this article^38^. Survey items were all obtained via self-report and included aspects of well-being such as happiness, health, meaning, character, relationships, and financial stability^41^ along with other demographic, social, economic, political, religious, personality, childhood, community, health, and well-being variables. The data are publicly available through the Center for Open Science (https://www.cos.io/gfs). During the translation process, Gallup adhered to TRAPD model (translation, review, adjudication, pretesting, and documentation) for cross-cultural survey research (ccsg.isr.umich.edu/chapters/translation/overview).

### Measures

Demographics Variables: We reclassified continuous ‘age’ as 18–24, 25–29, 30–39, 40–49, 50–59, 60–69, 70–79, and 80 or older. We assessed ‘gender’ as male, female, or other; ‘marital status’ as single/never married, married, separated, divorced, widowed, and domestic partner; and ‘employment’ as employed by others, self-employed, retired, student, homemaker, unemployed and searching, and other. We assessed ‘education’ as up to 8 years, 9–15 years, and 16+ years; and ‘religious service attendance’ as more than once/week, once/week, one-to-three times/month, a few times/year, or never. We assessed ‘immigration status’ with the questions: “Were you born in this country, or not?”. Religious tradition/affiliation was assessed using the categories Christianity, Islam, Hinduism, Buddhism, Judaism, Sikhism, Baha’i, Jainism, Shinto, Taoism, Confucianism, Primal/Animist/Folk religion, Spiritism, African-Derived, some other religion, or no religion/atheist/agnostic—although response categories varied by country^39^. We assessed ‘racial/ethnic identity’ in most countries, but response categories varying by country. For additional details on the assessments see the COS GFS codebook or Crabtree et al. (2021)^42^.

Outcome Variable: The survey asked respondents if they agreed or disagreed with the following statement: “I tell other people about my religion or spirituality even when they have different beliefs [Agree, disagree, not relevant, unsure]”. We recoded responses as ‘Agree’ = 1, with all other responses recorded as 0. We refer to this as ‘faith sharing’.

The GFS encouraged respondents who identified as ‘not religious’ to use the ‘not relevant’ category for questions about religion they did not think applied to them and most people who responded ‘not relevant’ on the ‘faith sharing’ question are ‘non-religious’. Adherents of East Asian religions were more likely to respondent ‘unsure’ than other religious respondents. However, if we drop all ‘unsure’ or ‘don’t know’ respondents from the analysis, results are virtually identical (see Supplemental Table S24).

Of course, telling others about one’s religion can have a range of meanings. It is possible to tell someone about your religion without intending to convert them. However, four factors cumulatively suggest that ‘faith sharing’ often involves attempted persuasion. First, in the 2008–2012 Pew Research Center’s Survey of 39 Muslim countries, 60.1% of Muslims said they “have a duty to try to convert others to [Islam]” which is almost identical to the 59% of Muslims in Wave 1 of the GFS who say they share their faith with others. This overlap is unlikely if most ‘faith sharing’ by Muslims was merely informational.

Second, the 2007 Baylor Religion Survey asked a nationally-representative sample of people in the US “if one wishes to be a good person” how important is it to “convert others to your religious faith?”; 33.3% said it was ‘very important’ or ‘somewhat important’ (13.4% & 19.9% respectively). This is roughly the same percent of US respondents on the GFS who report sharing their faith (37%). Both surveys asked the same question to all respondents, including the non-religious. Thus, the comparable percentages suggest conversion is important to most GFS ‘faith-sharers’ in the US.

Third, the 2014 Pew ‘U.S. Religion Landscape Survey’ asked all respondents who had a religious identity “How often do you share your faith with non-believers or people from other religious backgrounds—would you say at least once a week, once or twice a month, several times a year, seldom, or never?” (underline added). 54.8% of ‘religious’ respondents said they ‘shared their faith’ several times a year or more (i.e., 25.5% weekly, + 15.3% 1 or 2 × per month, + 14.0% several times a year = 54.8%). Among ‘religious’ respondents on the US sample of the GFS, 46.5% said they “tell other people about [their] religion or spirituality even when they have different beliefs”. The Pew question emphasizes conversion more than the GFS question, yet gets a higher percent saying they share their faith.

This comparability is not limited to Christians. If we compare followers of religious traditions other than Christianity, in the GFS’s US samples 42.8% share their faith and in the ‘U.S. Religious Landscape Survey’ 38.6% do. This suggests that the proselytism-component of ‘faith sharing’ is common for both Christians and non-Christians in the US.

Unfortunately, because questions about evangelism, conversion, religious persuasion, and missions are so rare on nationally representative surveys of adults (especially outside the US), we do not know of any other surveys we can use to compare with the GFS question. However, every survey we can match consistently suggests the percent of ‘share their faith’ matches the percent who think converting others is important, etc.

Fourth, the consistent strong correlation between religious service attendance and faith sharing in every country on the GFS suggests that respondents most committed to their tradition are more likely to share regardless of context. Still, in many contexts ‘faith sharing’ is probably trying to change the beliefs, denominational affiliation, or religiosity of co-religionists, e.g., a charismatic Catholic sharing about ‘speaking in tongues’ with a non-charismatic Catholic. Regardless, the term ‘faith sharing’ has sufficient ambiguity to cover both informational discussions and attempts to persuade.

### Statistical analysis

For each demographic variable, we estimated descriptive statistics for the full sample, with the data weighted to be nationally representative within each country. We estimated nationally representative proportions for ‘evangelism’ separately for each country and list them from highest to lowest with their 95% confidence intervals and standard deviations. We also estimated the proportion who ‘evangelize’ in each of the demographic categories in each country/territory (see Supplementary Material). We then conducted a random effects meta-analyses of the country-specific proportions of people who ‘evangelize’ for each demographic category^43,44^, along with 95% confidence intervals, standard errors, and upper and lower limits of the 95% prediction interval. For each response category, we measured variation across countries using the heterogeneity (τ), and I^2^ ^45^. Forest plots in the online supplement show the proportions and 95% confidence intervals for each country on each coefficient.

We conducted all meta-analyses in R^46^ using the metafor package^47^. Within each country, we conducted a global test of variation across levels of each demographic variable, and computed a pooled p-value^48^. The p-value indicates whether the proportion who ‘evangelize’ varies significantly between the response options for that variable in any of the countries in our sample. For example, in Table 3 the global p-value for age is < 0.001, which suggests that age is associated with the proportion of people who ‘evangelize’ in at least one country. We use Bonferroni corrected p-value thresholds based on the number of demographic variables considered in our analysis^45,49^. Because different countries have different religious affiliation/tradition and race/ethnicity categories, we do not include them in our meta-analysis. However, we measure them in the country specific analyses in Supplemental Tables S1aS22b & S1b-S22b. In the meta-analysis in the text (Table 3) each country/territory is weighted equally. In the supplementary material, we redo the meta-analysis with countries weighted by their population size. We pre-registered all our analyses with COS prior to data access (https://osf.io/8edcx); all code to reproduce analyses are openly available in an online repository^38^.

### Missing data

We imputed missing data for all variables using multivariate imputation by chained equations and five imputed datasets^50–53^. Because the categories for religious affiliation/tradition and race/ethnicity varied between countries, we imputed data separately for each country. This within-country imputation approach ensured that the imputation models accurately reflected country-specific contexts and assessment methods. We included sampling weights in the imputation model to account for specific variable missingness related to probability of overall study inclusion.

### Accounting for complex sampling design

The GFS used different sampling schemes across countries based on availability of existing panels and recruitment needs^38^. All analyses accounted for the complex survey design components by including weights, primary sampling units, and strata. Additional methodological detail, including accounting for the complex sampling design is provided elsewhere^40^.

## Results

Table 1 shows nationally representative descriptive statistics of the demographic characteristics in our sample (all 22 countries combined): 51% are female, 53% are married, 57% are employed, 57% have 9–15 years of education, 63% attend religious services, 94% are native to their country.

**Table 1:** Nationally-representative descriptive statistics of the observed sample.

Variable	Proportion	Frequency
Age		
18–24	0.13	27,007
25–29	0.10	20,700
30–39	0.20	40,256
40–49	0.17	34,464
50–59	0.16	31,793
60–69	0.14	27,763
70–79	0.08	16,776
80 or older	0.02	4119
Missing	0.00	20
Gender		
Male	0.49	98,411
Female	0.51	103,488
Other	0.00	602
Missing	0.00	397
Marital status		
Single/never been married	0.26	52,115
Married	0.53	107,354
Separated	0.03	5195
Divorced	0.06	11,654
Widowed	0.05	9823
Domestic partner	0.07	14,931
Missing	0.01	1826
Employment		
Employed for an employer	0.39	78,815
Self-employed	0.18	36,362
Retired	0.14	29,303
Student	0.05	10,726
Homemaker	0.11	21,677
Unemployed and looking for a job	0.08	16,790
None of these/other	0.04	8431
Missing	0.00	793
Education		
Up to 8 years	0.22	45,078
9–15 years	0.57	115,096
16 + years	0.21	42,578
Missing	0.00	146
Service attendance		
> 1/week	0.13	26,537
1/week	0.19	39,157
1–3/month	0.10	19,749
A few times a year	0.20	41,436
Never	0.37	75,297
Missing	0.00	722
Immigration status		
Born in this country	0.94	190,998
Born in another country	0.05	9791
Missing	0.01	2110
Country		
Argentina	0.03	6724
Australia	0.02	3844
Brazil	0.07	13,204
Egypt	0.02	4729
Germany	0.05	9506
Hong Kong	0.01	3012
India	0.06	12,765
Indonesia	0.03	6992
Israel	0.02	3669
Japan	0.10	20,543
Kenya	0.06	11,389
Mexico	0.03	5776
Nigeria	0.03	6827
Philippines	0.03	5292
Poland	0.05	10,389
South Africa	0.01	2651
Spain	0.03	6290
Sweden	0.07	15,068
Tanzania	0.04	9075
Turkey	0.01	1473
United Kingdom	0.03	5368
United States	0.19	38,312

*Country-specific descriptive statistics are available in the Online Supplement.

Supplemental Tables S1a-S22a show nationally representative descriptive statistics for each country. One striking difference between countries is the percent who ‘never attend religious service’ (from 1% in Nigeria to 77% in Japan; and the percent who say they are ‘Atheist, Agnostic, or have no religion’—*henceforth ‘the non-religious’*): which varies from 0% in Nigeria and Indonesia to 61% in Japan. The prevalence of the ‘non-religious’ and ‘never attenders’ seems to influence a national culture of ‘faith sharing’ (*see discussion below*).

Table 2 shows the ordered proportions of people ‘sharing their faith’ across countries. The mean level of sharing is 46% (i.e., the mean of national means). The highest percentage of people who share their faith are in Tanzania (83% share, 95% confidence interval [CI] 0.82, 0.85) and Kenya (81% share, 95% CI: 0.80, 0.83). The other top-ranked countries are Nigeria (76%), South Africa (72%), and India (70%). All have low GDPs per-capita, are majority Christian or Hindu, have high religious diversity, have few ‘nonreligious,’ and are former British colonies—and thus had high exposure to non-State Christian missions. Moreover, every sub-Saharan African or South Asian country in our sample is in the top five ‘faith sharing’ countries, and there is an 8 percentage point drop to the 6th country: i.e., in Brazil 62% share (95% CI: 0.61, 0.63).

**Table 2:** Ordered means/proportions for ‘sharing faith’ in each country.

Country	Mean/proportion	LCI	UCI	SD
Tanzania	0.83	0.82	0.85	0.37
Kenya	0.81	0.80	0.83	0.39
Nigeria	0.76	0.74	0.79	0.42
South Africa	0.72	0.70	0.75	0.45
India	0.70	0.69	0.71	0.46
Brazil	0.62	0.61	0.63	0.48
Turkey	0.60	0.57	0.63	0.49
Philippines	0.56	0.54	0.57	0.50
Indonesia	0.55	0.53	0.56	0.50
Argentina	0.50	0.48	0.51	0.50
Israel	0.47	0.43	0.51	0.50
Mexico	0.47	0.45	0.48	0.50
Egypt	0.41	0.39	0.43	0.49
United States	0.37	0.36	0.38	0.48
Hong Kong	0.32	0.30	0.35	0.47
Poland	0.30	0.28	0.32	0.46
Spain	0.26	0.25	0.28	0.44
United Kingdom	0.23	0.22	0.25	0.42
Australia	0.23	0.21	0.25	0.42
Germany	0.21	0.20	0.22	0.41
Sweden	0.19	0.18	0.20	0.39
Japan	0.04	0.04	0.04	0.19

At the bottom of the ‘faith-sharing’ ranking is every European society in our sample, plus Australia, and Japan. In Poland, 30% share, in Spain 26%, in the UK 23%, Australia 23%, Germany 21%, Sweden 19%, and Japan 4% (although the 95% confidence intervals for UK, Australia, and Germany overlap). All these societies have had state-financed religions. These societies further sub-divide along religious lines: Catholic-majority Poland and Spain share significantly more than Protestant-majority UK, Australia, Germany and Sweden, which share significantly more than Japan. The difference between Japan and others societies is particularly striking: 15 percentage points fewer Japanese share than Swedes.

Although the US has a high GDP per-capita and a large European-ancestry population, a higher percentage of people in the US share their faith 37% (95% CI: 0.36, 0.38). Majority-Catholic former Spanish and Portuguese colonies, Muslim-majority societies, Israel, and Hong Kong are also in the middle.

We should be cautious about interpreting the precise rankings. Sometimes the confidence intervals of neighbors overlap and question-wording may have different connotations in different languages (despite our dedicated effort to minimize this). Caution is especially important when comparing adherents of Abrahamic faiths (Judaism, Christianity and Islam) with practitioners of East Asian ‘religious traditions’ in Hong Kong and Japan. The word used for ‘religion’ in both societies was coined in the nineteenth century in response to Christian missions and originally included Buddhism, but excluded Confucianism, Taoism, Shinto, and folk religion. Moreover, East Asian religions are often less exclusive than Abrahamic faiths and people often participate in rituals from several ‘traditions’ without identifying as ‘belonging’ to any of them. Moreover, beliefs often function subconsciously (like grammar) rather than consciously^54–59^. In Hong Kong, religious identities seem to be clearer and beliefs more articulated than in Japan, perhaps because in Hong Kong 25% of the population are Christian, religious diversity is greater, and much of the population has attended Christian schools^60^. Thus, for example, most Japanese buy amulets, believe in *kami* (spirits) and/or *hotoke* (dead ancestors or buddhas), and pray at shrines for good luck, while simultaneously saying they are not religious^54,55,58^. In fact, about half of Japanese say ‘they are not religious, but do rituals’^54^.

However, despite these caveats, the general pattern is revealing. There is some evidence of a negative correlation between GDP per capita and sharing and a positive correlation between being a former British colony and sharing. There also seem to be regional differences: sharing is high in Africa and India, and low in Europe and Japan. It is less clear whether there is a strong correlation between dominant religion and sharing. There are 22 countries/territories in our sample. Countries with large Muslim populations are widely dispersed: Tanzania is 1st, Nigeria 3rd, Turkey 7th, Indonesia 9th, and Egypt 13th. Countries with large Catholic populations are also dispersed, Brazil is 6th, Philippines 8th, Argentina 10th, Mexico 12th, Poland 16th, and Spain 17th. Countries with large Protestant populations range from Kenya (2nd) to Sweden (21st). Majority-Jewish Israel is in the middle (47% share, ranked 11th). In general, in more religiously diverse societies a higher proportion reported ‘faith sharing’.

We will discuss this later, but there also seems to be a spillover effect. In highly devout societies, most people share their faith, even those who never attend religious services and do not identify as ‘religious’. Conversely, in more secularized societies, even many devout people do not share their faith.

Sharing is also high in at least some ‘religiously homogeneous’ societies like the Philippines (93% Christian) and Turkey (94% Muslim). Thus, ‘faith sharing’ is not just about converting others between broad religious categories. ‘Faith sharing’ can also be people discussing and promoting different organizations, beliefs, and practices within their own broad religious tradition.

Table 3 shows random meta-analytic effects of sharing faith by demographic category (giving each country an equal weight). The global p-values suggest all the demographic variables are associated with ‘faith sharing’ in at least one country, and they exceed the Bonferroni corrected threshold of p < 0.007. However, when we compare the pooled mean values for these coefficients (weighting each country equally), some of the differences in meta-analytic means are small. For age, gender, employment status, education, and immigration status, the confidence intervals of all categories overlap and the means are mostly close together. However, some differences are clear: people who have a domestic partner are less likely to share their faith (23% do). Married people are more likely to share their faith. The main factor associated with faith sharing is religious service attendance. Interestingly, 27% of those who never attend religious services share their religious and spiritual beliefs with others.

**Table 3:** Random effects meta-analysis of sharing faith proportions by demographic category.

Prediction Interval									
Variable	Category	Proportion	95% CI of Proportion	SE Analogue (CI Width/4)	LL	UL	Heterogeneity (τ)	I^2	Global p-value
Age group	18–24	0.45	(0.35,0.57)	0.05	0.04	0.81	0.26	97.8	<.001**
	25–29	0.45	(0.33,0.57)	0.06	0.03	0.85	0.28	98.1	
	30–39	0.44	(0.33,0.56)	0.06	0.04	0.83	0.28	98.0	
	40–49	0.45	(0.33,0.57)	0.06	0.04	0.85	0.29	98.1	
	50–59	0.45	(0.33,0.58)	0.06	0.03	0.86	0.30	98.3	
	60–69	0.45	(0.33,0.58)	0.06	0.04	0.85	0.31	98.3	
	70–79	0.44	(0.32,0.56)	0.06	0.04	0.86	0.28	98.1	
	80 or older	0.64	(0.37,0.84)	0.12	0.06	1.00	0.60	99.6	
Gender									< .001**
	Male	0.43	(0.33,0.55)	0.06	0.04	0.82	0.27	97.8	
	Female	0.46	(0.34,0.58)	0.06	0.04	0.84	0.29	98.1	
	Other	0.17	(0.02,0.68)	0.16	0.00	1.00	0.78	99.9	
Marital status									< .001**
	Married	0.46	(0.35,0.58)	0.06	0.04	0.84	0.29	98.1	
	Separated	0.40	(0.29,0.51)	0.06	0.02	0.81	0.27	98.0	
	Divorced	0.43	(0.32,0.54)	0.05	0.05	0.77	0.26	97.8	
	Widowed	0.44	(0.31,0.57)	0.07	0.05	0.83	0.32	98.5	
	Domestic partner	0.23	(0.11,0.43)	0.08	0.00	0.77	0.39	99.4	
	Single, never married	0.44	(0.32,0.55)	0.06	0.04	0.82	0.28	98.0	
Employment status									< .001**
	Employed for an employer	0.44	(0.33,0.56)	0.06	0.04	0.85	0.28	98.0	
	Self-employed	0.46	(0.35,0.58)	0.06	0.06	0.83	0.27	97.8	
	Retired	0.45	(0.33,0.57)	0.06	0.04	0.83	0.28	98.1	
	Student	0.44	(0.33,0.56)	0.06	0.04	0.84	0.28	98.0	
	Homemaker	0.46	(0.34,0.58)	0.06	0.03	0.85	0.29	98.1	
	Unemployed and looking for a job	0.44	(0.33,0.56)	0.06	0.03	0.82	0.28	98.1	
	None of these/other	0.41	(0.29,0.55)	0.06	0.04	0.82	0.31	98.4	
Education									< .001**
	Up to 8 years	0.46	(0.34,0.59)	0.06	0.03	0.84	0.30	98.2	
	9–15 years	0.44	(0.33,0.56)	0.06	0.04	0.85	0.28	98.1	
	16 + years	0.44	(0.34,0.54)	0.05	0.05	0.79	0.25	97.6	
Religious service attendance	> 1/week	0.76	(0.70,0.81)	0.03	0.39	0.90	0.12	94.3	< .001**
	1/week	0.63	(0.57,0.69)	0.03	0.28	0.84	0.14	93.8	
	1–3/month	0.53	(0.45,0.61)	0.04	0.12	0.78	0.18	95.7	
	A few times a year	0.40	(0.32,0.50)	0.04	0.05	0.72	0.21	96.9	
	Never	0.27	(0.18,0.38)	0.05	0.02	0.65	0.23	98.0	
Immigration status									< .001**
	Born in this country	0.44	(0.33,0.56)	0.06	0.04	0.83	0.28	98.0	
	Born in another country	0.45	(0.33,0.59)	0.06	0.04	0.91	0.31	98.4	

*p < .05; **p < .007 (Bonferroni corrected threshold).

The τ shows how much the proportion of people who ‘shared their faith’ in that category varies across countries. For most variables, the τ is similar between categories. However, for respondents who are over 80, it is larger (0.60) than for other age groups (which range from 0.31 to 0.26).

If we recalculate the meta-analyses in Table 3 with each country weighted by its population size (which means India has about 40% of the influence and the five European countries only about 7%), the number of response options that are clearly distinct increases (Supplemental Table S23). The response options for gender and immigration status still have overlapping confidence intervals. However, people over 80 years old are more likely to share their faith than all younger groups. Married people are more likely to share, and both widows and those with a domestic partner are less likely to share. Self-employed people are more likely to share and students less likely to. Those with less than 8 years of education are less likely to share, and those who attend religious services regularly are more likely to share. The pattern of coefficients in Table S23 mirrors Table 3, but more differences are statistically significant.

Supplementary Tables S1b-S22b create the equivalent of Table 3 for each country. We discuss variables with a global p-value of p < 0.05, but only explicitly contrast response options if the 95% confidence interval of one does not overlap with the mean of the other, though this is a very conservative approach.

The most striking feature of the results is how much the patterns differ across countries. This explains why some of the pooled-mean differences in Table 3 are small. For example, in five countries younger people are more likely to share their faith (Egypt, Indonesia, Israel, Sweden, and the UK); in eight countries older people are more likely to share (Hong Kong, Spain, South Africa, Nigeria, Argentina, Kenya, Mexico, and Poland); in Turkey, Germany, and the US both younger and older people are more likely to share than the middle aged; in Brazil, middle-aged people are more likely to share than the old or young; in four countries, the differences are insignificant (India, Japan, Tanzania, and the Philippines), and one has no clear pattern (Australia).

The association with gender is more consistent. In ten countries, women are more likely to share their faith (Argentina, Australia, Brazil, India, Kenya, Nigeria, Poland, South Africa, and the US), and in all other countries there is little evidence for any gender difference.

There are significant differences between marital statuses in *all but six* countries (Germany, Japan, the Philippines, South Africa, Spain, and Tanzania). In most societies, married people are more likely to share and people with a domestic partner are less likely to share. Other marital statuses are less consistently associated with sharing.

Employment status is significantly associated with sharing in *all but eight* countries (Australia, Israel, Nigeria, the Philippines, South Africa, Spain, Tanzania, and Turkey). Generally, homemakers and the self-employed are more likely to share and students are less likely to share, but this is not always true. In Brazil and the UK, homemakers are significantly *less likely* to share, and in Egypt, Germany, the UK, and Indonesia, students are *more likely* to share.

The association between education and sharing also varies by countries. In six countries, education is unrelated to sharing (Australia, Brazil, Egypt, Nigeria, Poland, and Spain); in six countries, ‘more educated people’ share their faith more (Germany, Indonesia, Japan, the Philippines, Sweden, and the UK), in one the moderately educated share more (Turkey), and in nine the ‘less educated’ share more (Argentina, Hong Kong, India, Israel, Kenya, Mexico, South Africa, Tanzania, and the US).

The only completely consistent factor is religious service attendance. In every country those who attend religious services more often are more likely to share their faith.

Immigration status has a more complex association with sharing. In eleven societies, there is no association; in four societies, the native-born share more (Brazil, Israel, the Philippines, and the US) and in six, immigrants share more (Australia, Indonesia, Spain, Sweden, Tanzania, and the UK). In societies with lower levels of religiosity, the association is either insignificant or immigrants share more.

Type of religion is also associated with sharing in most societies. We only discuss groups that had a minimum of 30 respondents and made up at least 1% of the national sample. Typically Muslims, Christians, Hindus, and religious minorities were more likely to share their faith, and the non-religious were least likely to. However in the Philippines and Germany, Christians were in the group least likely to share.

The racial and ethnic categories vary radically between countries and so are hard to describe succinctly. Four countries did not ask about race or ethnicity, and in four countries the association is insignificant (Argentina, Brazil, Israel, and Mexico). Elsewhere, the only consistent pattern we noticed is that European-ancestry people are less likely to share their faith. Interestingly, in Hong Kong, even the provenance of ones’ Chinese ancestors matters, with those from Shanghai being the most likely to share – perhaps because of the historic prevalence of Christian missionaries there.

The forest plots (Supplementary Figures S1-S34) reveal which countries have proportions above or below the meta-analytic means. Generally, countries that have ‘higher-than-mean proportions’ are consistent, and likewise those that have ‘lower-than-mean proportions’ are consistent. Typically, in each category of each variable, African, southern Asian, and Latin American countries share faith more, and European countries and Japan share less. However, sometimes the position of particular countries changes radically between categories.

The most interesting country-shifts are for religious service attendance (Figures S28-S32). The positive association between religious service attendance and ‘sharing’ seems to be weaker for Indonesia, and stronger for Hong Kong and Sweden. At the lowest levels of attendance, Hong Kong and Sweden are towards the bottom of the plot, pulling the meta-analytic mean proportion who ‘share their faith’ towards zero, whereas Indonesia is near the top, pulling the proportion ‘sharing’ away from zero. However, at the highest levels of attendance, Hong Kong and Sweden are towards the top of the plot, pulling the proportion ‘sharing’ away from zero, and Indonesia is near the bottom pulling the proportion ‘sharing’ towards zero.

Forest plots also revel the dispersion in national means. The dispersion in ‘sharing’ increases as religious-service attendance decreases, i.e., the confidence interval almost doubles (from 0.11 to 0.20) and the range increases by 10 percentage points (with Japan in the sample) and 20 percentage points without Japan in the sample (see Figures S28 & S32).

There also seems to be a spillover effect between society-level religiosity and sharing. In societies where most people are devout, most people share their faith even if they attend religious-services irregularly or are ‘non-religious’. Whereas in more secularized societies, few people share their faith even if they are personally devout. For example, in Tanzania 89% of those who ‘attend more than one a week’ and 63% of those who ‘never attend’ share their religious beliefs with others. Whereas in Japan, 38% of ‘more than weekly attenders’ share compared to 2% of ‘never attender’ and in Germany 56% of ‘more than weekly attenders’ share compared to 14% of ‘never attenders’ (Supplemental Tables S10b & S19b). Thus, the least devout Tanzanians ‘share’ more than both the most devout Japanese or German and the mean ‘sharing’ in 17 countries. Moreover, the correlation between the ‘percent nonreligious’ in a country and the ‘percent who share their faith’ is −0.82, and the correlation between the ‘percent nonreligious’ in a country and the ‘the percent of atheists/agnostics/non-religious who share their faith is −0.77.

## Discussion

Using data from a diverse, international sample of 202,898 individuals across 22 countries, we examined the distribution of ‘faith sharing’ between countries by age, gender, marital status, employment status, religious service attendance, education, immigration status, race/ethnicity, and religious affiliation. Except for in a few countries like Japan, faith sharing is widespread. The mean level of ‘faith sharing’ is 46% (with each country weighted equally). In countries like Tanzania, 83% of people report ‘sharing their faith’ and even most ‘atheist, agnostic, or non-religious’ and most who ‘never attend religious services’ report sharing their faith (71% and 63% respectively).

Of course there are some limitations to our study. The survey asked people to respond to the following statement: “I tell other people about my religion or spirituality even when they have different beliefs [Agree, disagree, not relevant, unsure]” and 57.5% of ‘non-religious’ people answered ‘Not relevant.’ Only 16.2% of those who claimed a religious identity responded “not relevant.” However, this does not mean the ‘non-religious’ do not tell either ‘religious people’ or ‘other non-religious people’ their ‘non-religious beliefs,’ or try to convince ‘religious people’ or ‘other non-religious people’ to think more like they do. The ‘non-religious’ merely think the question does not apply to them. On the 2007 *U.S. Religious Landscape Survey,* 67.6% of ‘atheists, agnostics, and non-religious’ said they share their “views of God and religion with religious people.” Whereas in the U.S. sample of Wave 1 of the *Global Flourishing Study* (GFS) only 16% of ‘atheists, agnostics, and non-religious’ said they tell others “about my religion and spirituality.” The 51 percentage point difference between these two nationally representative U.S. surveys is similar in magnitude to the 57.5% of the ‘non-religious’ on the GFS who said the question did not apply to them.

Our results also reveal several theoretically important insights. First, ‘faith sharing’ is widespread in most religious traditions. If we analyze the percent of respondents from each religion who share their faith, Umbanda, Spiritists, and Hindus ‘share’ the most. However, they are widespread only in a few countries where ‘faith sharing’ is high. If we look for consistent patterns between countries, Hindus, Muslims, Christians, and religious minorities are consistently near the top. Yet, India and many Muslim-majority countries have high social and legal restrictions on ‘faith sharing’ by non-Hindus and non-Muslims respectively^61–63^.

Second, both European societies and people with European ancestry regardless of society, are less likely to share their faith. This may be a reaction to European colonialism and ethnocentrism, or because of greater secularism, or because of a history of state-funded religion. Conversely, in former colonies and places that received many Catholic and Protestant missionaries, faith-sharing is widespread.

Third, it is dangerous to generalize about ‘faith sharing’ from the US or other Christian-majority samples. Almost all research about evangelism, missions, and faith sharing focuses on the US. Yet, the demographic factors that predict faith sharing in the US do not generalize well. For example, in the US the less well educated ‘share’ more, but that is not true in most other societies.

In fact, it is dangerous to pool all countries without also looking at each country individually. In Table 3, which pools all countries, almost nothing seems to clearly predicts ‘faith sharing’ and the 95% confidence intervals often overlap between the response options. However, in spite of this, the global p-values are all highly significant (suggesting these factors matter in at least one country in the sample). Moreover, in each country-specific table, most demographic factors predict faith sharing. However, because the patterns are often opposite across countries, they cancel each other out in the pooled analyses. For example, in some societies the more educated share more and in a roughly equal number of societies the less educated share more. Thus, we need to pay particular attention to the cultural, historical, and political context in each society.

In spite of the variation, some demographic factors *often* predict faith sharing. Generally, married people share more and those with domestic partners less. This makes sense because many religions promote marriage and sanction extra-marital sexual relations. Similarly, homemakers and the self-employed often share more, and students less. This may be because students have more exploratory religious lives. Women often share their faith more than men, which makes sense because in most (but not all) societies women are more religiously active then men.^64^ The only completely consistent factors are that those who attend religious services more ‘share’ more, and those have a religious identity share more than those who are ‘non-religious.’

Fourth, there seems to be a spillover effect of religion. In societies with a high percent ‘non-religious’ and/or a high percent who ‘never attend’ religious services, the devout share infrequently. Conversely, in devout societies, even the ‘non-religious’ and those who ‘never attend’ often share frequently. It is tempting to speculate about why this pattern exists and at least three theories may explain it:

(1) Faith sharing may be one form of religious vitality. In countries where religion is thriving, people share. In societies where it is atrophying, they do not. Religion could be atrophying in European-ancestry societies because of high ‘existential security’ or other reason^65,66^.

(2) Alternatively, ‘faith sharing’ could be associated with religious markets and the recentness of religious change. For example, in much of Sub-Saharan Africa the spread of Christianity and/or Islam is very recent, i.e., mostly in the twentieth century^8^. Thus, religious organizations are young, entrepreneurial, recently connected to missions, and generally not state supported. Moreover, GFS survey respondents, their parents, or their grandparents are often the first generation of ‘believers.’ Thus, religious change may be seen as ‘normal.’ Conversely, in societies were the adoption of the dominant religious tradition happened centuries or millennia ago and where the state has supported the dominant religion and restricted alternatives, religious sharing may seem offensive.

(3) Pierre Bourdieu argues that elites shape the types of behavior, tastes, and opinions that either confer status or are ‘polluting,’ i.e., his theory of ‘social capital’ and ‘fields.’ Moreover, intellectuals and other elites distinguish themselves from ordinary people by constructing ‘symbolic boundaries’^67,68^. In Europe and North America, elites often use ‘non-religion’ or ‘eclectic spirituality’ as a symbolic boundary with ordinary people^4,13,69–71^. This may make public ‘faith sharing’ gauche. Conversely, in non-Western societies elites may use other symbolic boundaries, and many elites graduated from mission schools, etc. Perhaps in these contexts ‘faith sharing’ is not gauche. Countries like Singapore and South Korea would be ideal to test these theories, because they have high GDPs, high Human Development, widespread recent religious change, and educated elites that are disproportionately graduates of mission schools^72–76^.

However, adjudicating between these theories is beyond the scope of this paper. As more waves of GFS data become available, we can increasingly test causal arguments.

To our knowledge, this is the first study of ‘faith sharing’ in a large, global, multi-religious sample of countries. We hope it spurs future research. In a world where restrictions of religious liberty are widespread, violence against religious minorities endemic, and religious double standards prevalent, it is important to provide a mirror that consistently compares religious traditions about issues like ‘faith sharing’ that most do, but often simultaneously resent in others.

## Supplementary Information

Supplementary Information.

## Data Availability

The data are publicly available through the Center for Open Science (https://www.cos.io/gfs). All analyses were pre-registered with COS prior to data access (https://osf.io/dv2mq/?view_only=c415b4dbbe394b6eb5707b26c170deb4); all code to reproduce analyses are openly available in an online repository.
